# PEG-associated efficacy loss in lipid nanoparticles persists across formulation modifications for mRNA delivery

**DOI:** 10.21203/rs.3.rs-9763025/v1

**Published:** 2026-06-09

**Authors:** Mariah L. Arral, Namit Chaudhary, Katherine C. Fein, Alexandra N. Newby, Saigopalakrishna S. Yerneni, Jilian R. Melamed, Samuel T. LoPresti, Daria M. Strelkova Petersen, Kathryn A. Whitehead

**Affiliations:** Carnegie Mellon University; Carnegie Mellon University; Carnegie Mellon University; Carnegie Mellon University; Carnegie Mellon University; Carnegie Mellon University; Carnegie Mellon University; Carnegie Mellon University; Carnegie Mellon University

**Keywords:** Lipid nanoparticles, mRNA delivery, PEG, immune responses

## Abstract

Lipid nanoparticles (LNPs) are clinically proven mRNA delivery vehicles, and repeated administration is often required to maintain therapeutic or prophylactic benefit. However, a single dose can provoke adaptive immune responses against polyethylene glycol (PEG), a polymeric excipient on the LNP surface. In this event, resultant anti-PEG antibodies neutralize subsequent LNP doses, causing efficacy loss and limiting therapeutic utility. Here, we investigated whether altering the LNP formulation between initial and subsequent doses could mitigate PEG-associated efficacy loss. Following the administration of an LNP known to elicit anti-PEG antibodies, we re-dosed with LNPs formulated with varied PEG-lipid chemistry, PEG concentration, mRNA cargo, and administration route. None of these adjustments prevented efficacy loss, which consistently correlated with elevated anti-PEG immunoglobulin M (IgM) and, in some cases, immunoglobulin G (IgG). Reduced PEG concentration diminished immunogenicity but also potency, and intraperitoneal dosing elicited the strongest responses upon repeat injection. Further, LNPs that elicit anti-PEG antibodies also impaired the subsequent delivery of an FDA-approved MC3 formulation, suggesting that anti-PEG immune responses can compromise any PEGylated LNP formulation. Together, these findings demonstrate that PEG-associated efficacy loss cannot be resolved through formulation optimization alone. Instead, they suggest that the ionizable lipid’s engagement of innate immune receptors is the primary determinant of repeat dosing success.

## Introduction

Lipid nanoparticles (LNPs) are the most clinically advanced non-viral delivery vehicles for RNA, given the success of patisiran and COVID-19 mRNA vaccines[1–3]. Beyond vaccination[4, 5], LNPs hold promise for diverse therapeutic applications, including protein replacement [6–8], gene therapy[9, 10], and immunotherapy [11–13]. Standard LNPs comprise four lipids: an ionizable lipid, a sterol, a phospholipid, and a polyethylene glycol (PEG)-lipid, with each influencing formulation stability, biodistribution, and intracellular delivery[14].

Although PEG-lipids represent only 1–3 mol% of typical LNP formulations, they exert outsized effects on colloidal stability[15], particle size[16], protein adsorption[17], and immune shielding[18]. PEG-lipids consist of a PEG polymer conjugated to a lipid anchor, and the molecular weight of the PEG chain and the length of the lipid tails can each be varied. The lipid anchor incorporates non-covalently into the nanoparticle surface and, once administered in vivo, gradually dissociates through PEG shedding, with shorter lipid tails resulting in faster dissociation[19]. PEG concentrations must be carefully balanced, as higher concentrations prolong circulation but can also hinder cellular uptake[19]. Currently, FDA-approved PEG-lipids include PEG2000-C-DMG, DMG-PEG2000, and ALC-0159, and lipid nanoparticles formulated with PE-PEG2000 (C14-PEG2000) are frequently reported in the literature [20–24].

Because mRNA expression is transient, repeated administration is typically required for therapeutic effect[25]. This includes not only prime-boost vaccination regimens but also chronic protein replacement therapies and gene-modulating treatments, where redosing is intrinsic to clinical utility[14]. However, repeat dosing is not possible for some effective LNP formulations, with efficacy loss being linked to anti-PEG antibodies[26–30]. This is consistent with the phenomenon of accelerated blood clearance, which was first described in PEGylated liposomes[29]. We recently demonstrated that, in the case of LNPs, failed repeat dosing can be attributable to specific ionizable lipids that do not bind the innate immune receptors TLR4 and CD1d[30, 31]. Lack of innate immune system engagement results in anti-PEG IgM production, leading to efficacy loss upon repeat dosing. Much remains to be understood regarding the mechanism by which the immune system recognizes LNPs and prevents successful repeat administration[30].

Here, we investigate whether formulation optimization can restore repeated dosing performance for an LNP that exhibits PEG-associated efficacy loss. We hypothesized that anti-PEG antibodies would recognize any altered formulation that contained some form of PEG. Indeed, this was the case regardless of PEG chemistry, mRNA cargo, formulation ratios, and administration route. Furthermore, our data indicate that pre-existing anti-PEG antibody levels inhibit *de novo* efficacy of lipid nanoparticles formulated with FDA-approved ionizable lipid MC3, which is normally capable of repeat dosing. Together, these findings establish that PEG-associated efficacy loss is a consequence of ionizable lipid immune effects and that ionizable lipid selection, not PEG-lipid modification, is critical in achieving durable repeat dosing performance.

## Results and Discussion

Recently, we reported that the ionizable lipid 304O_i10_ fails to engage the innate immune receptors TLR4 and CD1d, resulting in uninhibited anti-PEG IgM production and loss of delivery efficacy upon repeat dosing[30]. Here, we asked whether modifying other formulation parameters could rescue repeat dosing performance despite this innate immune deficit. To address this, C57BL/6 mice received injections of LNPs 14 days apart, with some experiments including a third dose on day 28. LNPs were loaded with either firefly luciferase or human erythropoietin (EPO), with efficacy being evaluated 6 hours post-injection by IVIS or serum ELISAs, respectively. To monitor the humoral immune response, we evaluated IgM, anti-PEG IgM, IgG, and anti-PEG IgG expression. Blood draws were timed to capture antibody kinetics relative to each dose (Scheme 1) [32]. Day – 1 establishes a baseline, and day 7 captures the early T-independent IgM response to the first dose [30]. Then day 13 establishes the pre-second-dose baseline, and day 21, which is 7 days after the second dose, was chosen to detect any boost or class switching from repeat exposure. Finally, day 28 monitors persistence two weeks after the second dose [30].

### Efficacy loss persists across mRNA cargos and correlates with anti-PEG antibody production

Because mRNA therapies may employ different payloads across indications, we first asked whether mRNA cargo identity influences the severity of PEG-associated efficacy loss. To test this, we employed an LNP comprising the ionizable lipid 304O_i10_, cholesterol, DOPE, and PE-PEG2000 at the molar ratio of 35:46.5:16:2.5 as described previously[30]. We chose the ionizable lipid 304O_i10_ for these studies because we have previously demonstrated that LNPs containing this lipid lose delivery efficacy upon repeat dosing due to lack of engagement of innate immune receptors and resultant uninhibited anti-PEG IgM production[30].

**Figure 1:**
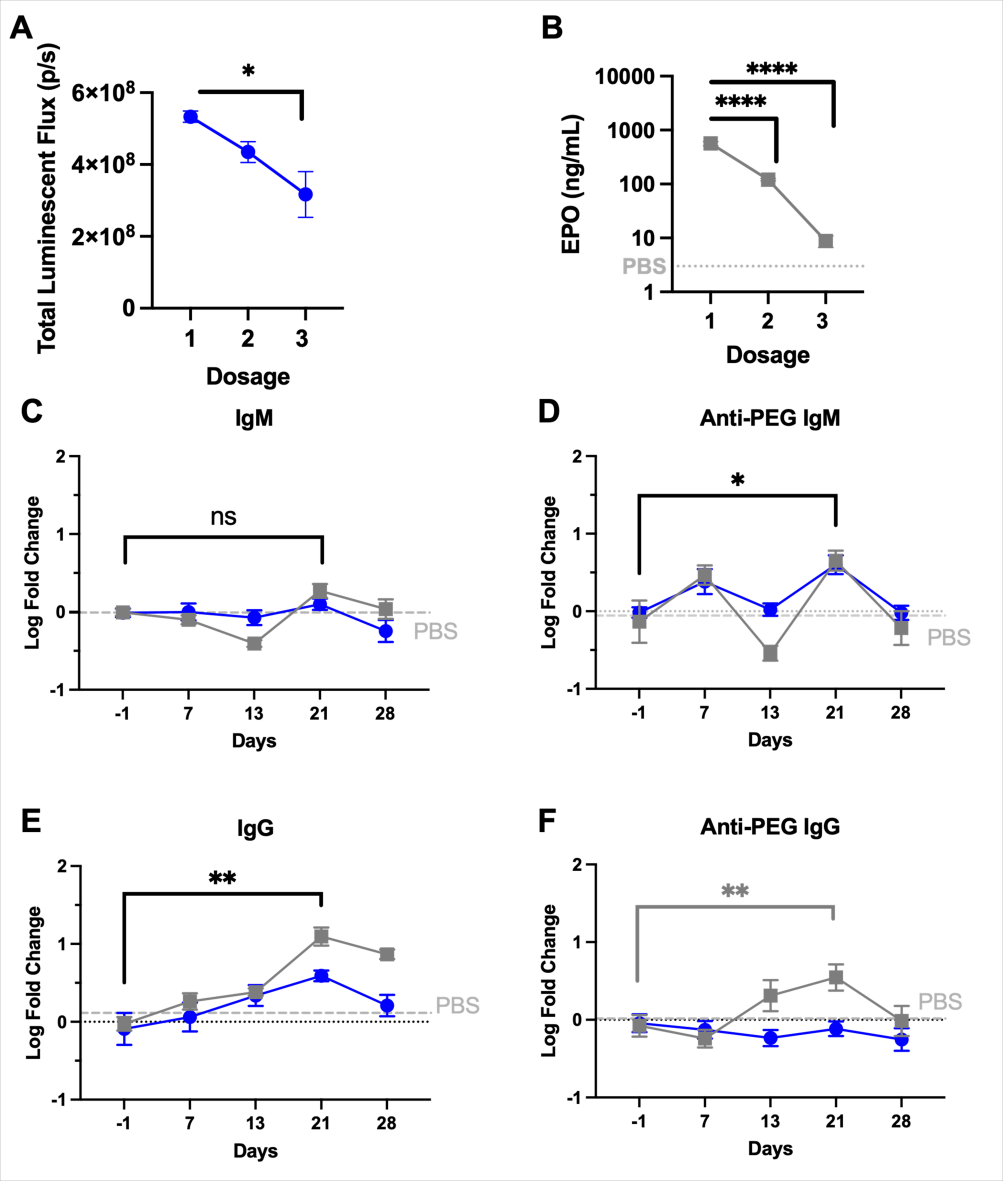
Regardless of the mRNA cargo, 304O_i10_ LNPs lose efficacy upon repeat administration. Lipid nanoparticles formulated with 304O_i10_ and mRNA encoding either A) firefly luciferase (blue circles) or B) human erythropoietin (EPO, gray squares) were delivered IV to mice at 0.5 mg/kg total mRNA. Animals were re-dosed twice at two-week intervals. At 6 hours post-injection, EPO concentration was determined by ELISA, and luciferase expression was quantified by IVIS imaging. Blood was collected weekly for 4 weeks, and C) IgM, D) Anti-PEG IgM, E) IgG, F) Anti-PEG IgG serum levels were quantified by ELISA. The gray dashed line indicates the PBS control serum values. Anti-PEG IgM and total IgG levels increased significantly on day 21 compared to day −1. Anti-PEG IgG increased significantly for EPO only. N=3–4, error bars represent SEM, with * and **** indicating P < 0.05 and P < 0.0001, respectively, by two-way ANOVA.

Initial studies used LNPs encapsulating firefly luciferase mRNA, administered intravenously (IV) at 0.5 mg/kg on a three-dose schedule, as in Scheme 1. Luciferase expression decreased with each administration, with the third dose producing less than half the signal of the first (Fig. 1A). To test whether efficacy loss depended on the encoded protein, we swapped luciferase mRNA for EPO mRNA, a mammalian protein natively expressed in hepatocytes[33–35]. Notably, EPO expression was more strongly dependent on repeat dosing; by the third dose, EPO serum levels declined ~ 80-fold relative to the first (Fig. 1B). These results demonstrate that efficacy loss occurs independent of the specific mRNA sequence or size, consistent with prior reports attributing efficacy loss to PEG-lipid components[27]. However, mRNA identity may influence the magnitude of that loss.

Prior studies have linked this efficacy loss phenomenon to anti-PEG IgM production, including for 304O_i10_ LNPs [26, 30]. We therefore measured total IgM, anti-PEG IgM, total IgG, and anti-PEG IgG after each dose. An increase in nonspecific IgM marks general inflammation[36], while elevated nonspecific IgG indicates mature naïve B cell activation and class switching[26, 37]. Increases specific to anti-PEG IgM or IgG implicate the PEG-lipid as the immunogen. Tracking both isotypes is therefore important in defining PEG-driven immunogenicity [26, 37]. As expected, total IgM remained unchanged from day – 1 to day 21 (Fig. 1C). Anti-PEG IgM increased in both cohorts, but the magnitude and kinetics differed: luciferase mRNA-loaded LNPs elicited only a minimal rise between days 13 and 21, whereas EPO mRNA-loaded LNPs produced a 5-fold increase (Fig. 1D). Total IgG levels rose for both reporters, with 14- and 4-fold increases for EPO LNPs and luciferase LNPs, respectively (Fig. 1E). Notably, only EPO LNP dosing produced a significant rise in anti-PEG IgG (Fig. 1F).

Collectively, our data indicate that PEG-specific antibody responses consistently emerge upon 304O_i10_ LNPs administration. The magnitude and isotype distribution of these responses vary with the encoded mRNA and correspond with functional efficacy loss. This suggests that the mRNA payload influences the severity of PEG-driven adaptive immunity. Because luciferase and EPO differ in multiple respects, including mRNA length, codon composition, and whether the encoded protein is foreign or endogenous, we cannot distinguish which factor drives the stronger antibody response for EPO LNPs. One possibility is that EPO protein itself, though natively expressed, may be presented at supraphysiological levels that provoke conventional antigen presentation alongside the PEG-directed response. Regardless of mechanism, these data indicate that cargo selection is a variable that should be controlled in repeat dosing studies.

Regarding adaptive immunity mechanisms, the early rise in anti-PEG IgM without changes in total IgM suggests a PEG-specific, T-independent B-1 cell response, consistent with prior work on PEGylated liposomes [26]. The subsequent induction of IgG reflects class switching and B-2 cell involvement, indicative of a maturing adaptive response. Together, these results indicate that PEG-lipid exposure consistently provokes antibody production, with IgG kinetics closely tracking with the severity of efficacy loss.

### Administration route influences the magnitude but not the occurrence of efficacy loss

The accelerated clearance of PEGylated nanoparticles has been studied primarily after intravenous injection[27], and the influence of administration route on efficacy loss remains unclear. Understanding this is important because administration route determines the immune cell populations first exposed to LNPs[14]. To address this, we delivered 304O_i10_ LNPs intravenously (IV), intramuscularly (IM), or intraperitoneally (IP) on a two-dose schedule, measuring serum EPO levels 6 hours after each administration and monitoring antibody levels longitudinally.

**Figure 2:**
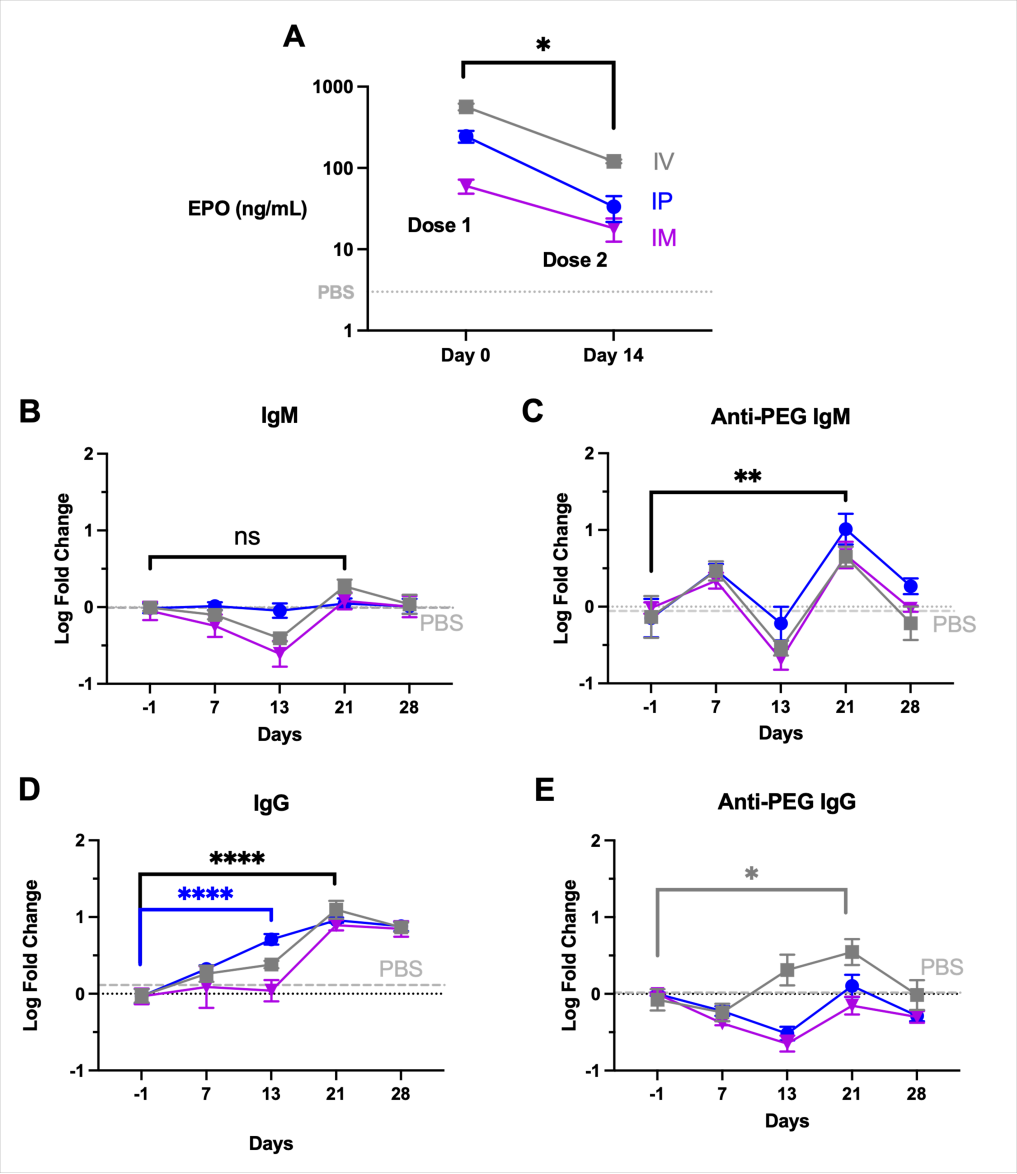
Regardless of administration route, 304O_i10_ LNPs lose efficacy upon repeat administration. Lipid nanoparticles formulated with 304O_i10_ and mRNA encoding EPO were delivered intravenously (IV, gray squares), intramuscularly (IM, purple triangles), or intraperitoneally (IP, blue circles) to mice at 0.5 mg/kg. Two doses were given 14 days apart. A) Serum EPO concentrations were quantified by ELISA 6 hours after each dose, and efficacy decreased with each dose, regardless of route of administration. (B-E) Serum antibody levels were quantified over time by ELISA. Anti-PEG IgM and total IgG increased significantly on day 21 compared to day −1, with total IgG for IP increasing significantly as early as day 13. Anti-PEG IgG increased significantly for IV only. The gray dashed line indicates serum PBS control values. N=3–4, error bars represent SEM, with * and *** indicating P < 0.05 and P < 0.001, respectively, by two-way ANOVA.

As is common with LNPs[38], the potency of 304O_i10_ LNPs varied by route, with intravenous delivery producing the highest EPO concentrations and intramuscular the lowest (Fig. 2A). Despite these differences, all routes showed significant efficacy loss upon repeat dosing. IV and IM injection each produced a ~ 4.7-fold reduction in EPO expression, whereas IP delivery produced a ~ 9.7-fold reduction. These results demonstrate that PEG-associated efficacy loss is a generalizable phenomenon independent of administration route.

Antibody profiling after a second dose revealed a consistent link between efficacy loss and anti-PEG responses. Total IgM levels remained unchanged across routes (Fig. 2B), whereas anti-PEG IgM increased significantly from day – 1 to day 21, with the largest fold increase (13x) observed after IP dosing (Fig. 2C). Total IgG levels also rose steadily (Fig. 2D), and only IV delivery produced a significant increase in anti-PEG IgG (4x, Fig. 2E). Notably, IP delivery produced a significant increase in total IgG as early as day 13, earlier than the other routes. The limited induction of anti-PEG IgG across most administration routes further supports the conclusion that PEG-associated efficacy loss is mediated primarily by early IgM responses rather than by mature, class-switched immunity. This distinction is critical. Strategies aimed solely at suppressing IgG formation are unlikely to preserve repeat dosing performance.

The enhanced efficacy loss observed following IP delivery is consistent with direct exposure of PEGylated LNPs to immune cells in the peritoneal cavity, a compartment enriched in macrophages and B cells that enables rapid immune recognition without nanoparticle extravasation from the vasculature[26, 39]. Consistent with this interpretation, IP administration produced the largest induction of anti-PEG IgM, coinciding with the greatest reduction in protein expression. The earlier onset of total IgG elevation following IP delivery further supports accelerated immune activation in this compartment. Recent work demonstrating macrophage-mediated gene transfer following IP LNP exposure underscores the heightened immune engagement in this route [39]. Although IP administration is less clinically advanced than IV or IM delivery, it provides a more sensitive model of immune surveillance, with increased immune cell contact amplifying PEG-associated efficacy loss.

### PEG-lipid chemistry alters antibody magnitude without preventing efficacy loss

Having demonstrated that efficacy loss occurs regardless of administration route, we next asked whether altering PEG-lipid chemistry could mitigate it. PEG-lipid variants differ in linker chemistry and PEG chain length, both of which influence shedding rate, surface PEG density, and potentially epitope accessibility[14, 40]. For these experiments, we compared repeat dosing performance for LNPs formulated with three commonly used PEG-lipids: phosphoethanolamine (PE)-PEG2000, PE-PEG750, and DMG-PEG2000 (**Fig S2**). PE-PEG2000, which has a PEG molecular weight of 2,000 g/mol, is frequently used in experimental mRNA formulations [20–24], whereas DMG-PEG2000 is used in the Moderna COVID-19 vaccine[41]. In addition to linker composition (PE vs. glycerol in DMG), we tested the effect of PEG chain length by including PE-PEG750, which is less than half the molecular weight of PE-PEG2000.

**Figure 3:**
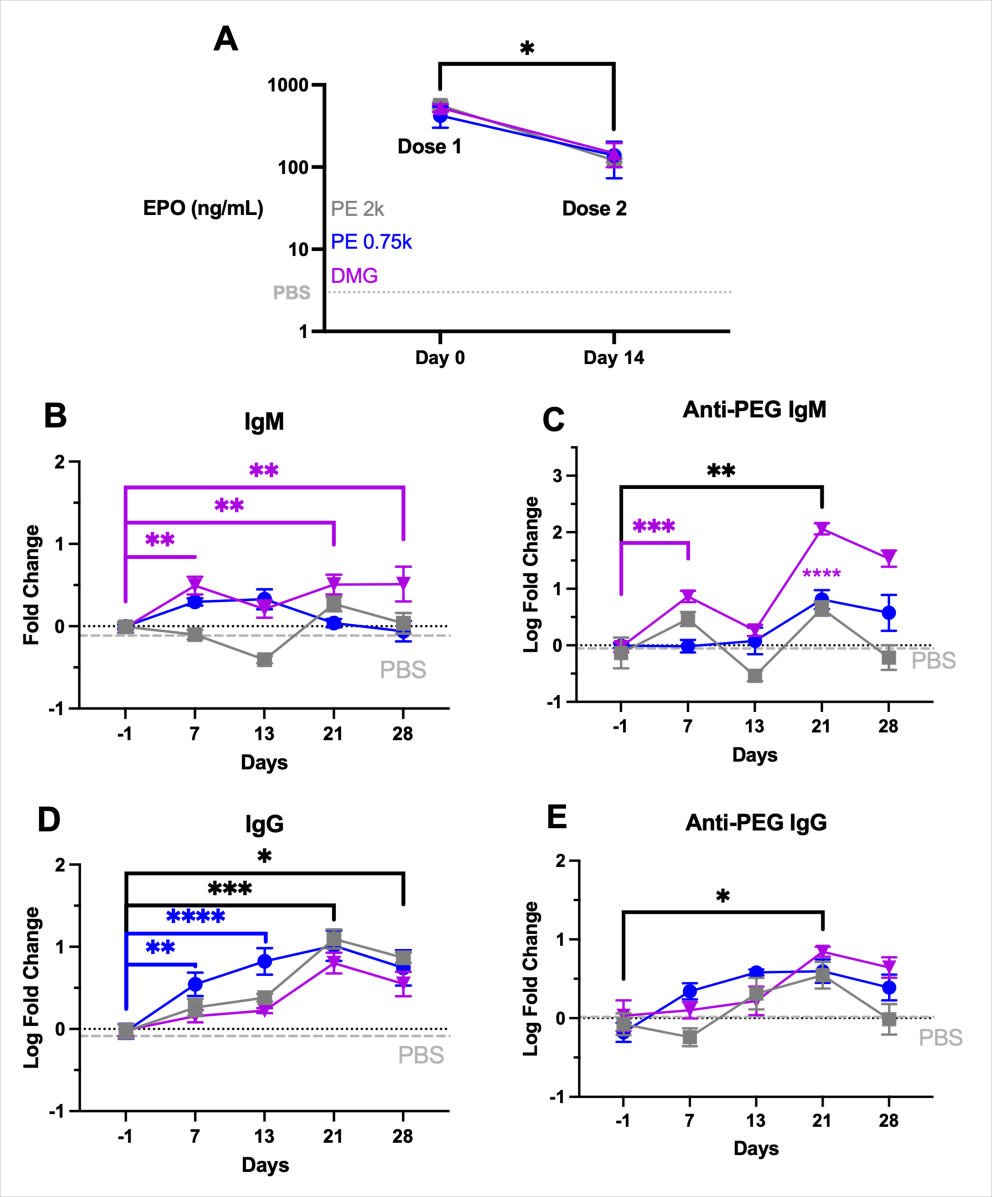
PEG-lipid chemistry modulates immune response magnitude but not efficacy loss. Lipid nanoparticles formulated with 304O_i10_ and one of PE-PEG2000 (gray squares), PE-PEG750 (blue circles), or DMG-PEG2000 (purple triangles) were delivered IV to mice at 0.5 mg/kg with mEPO. Serum EPO concentrations were quantified by ELISA 6 hours after each dose and decreased with each dose, regardless of PEG type. (B-E) Serum antibody levels were quantified over time by ELISA. Anti-PEG IgM, total IgG, and anti-PEG IgG increased significantly for all groups on day 21 compared to day −1. Total IgM increased significantly for DMG only. The gray dashed line indicates the serum levels of the PBS control group. N=3–4, error bars represent SEM, with * and **, ***, **** indicating P < 0.05, and P < 0.01, P < 0.001, P < 0.0001, respectively, by two-way ANOVA.

All PEG-lipid formulations produced nearly identical reductions in EPO expression after repeat dosing, confirming that efficacy loss persists across PEG-lipid variants (Fig. 3A). Potency across formulations was comparable, with fold decreases in EPO ranging from 3.1 to 4.7. Thus, neither PEG chain length nor linker chemistry prevented adaptive immune responses.

In contrast, antibody responses differed across formulations. DMG produced the strongest anti-PEG IgM induction, with a 125-fold increase by day 21, compared to 5-fold and 8-fold increases for PE-PEG2000 and PE-PEG750, respectively (Fig. 3C). Only DMG elevated total IgM, suggesting an inflammatory response (Fig. 3B). This may reflect the lipid anchor chemistry of DMG, which contains a glycerol linker rather than the phosphoethanolamine group, or interactions between DMG-PEG2000 and 304O_i10_ that influence innate immune activation. As with the formulations tested in Fig. 2, total IgG increased across all groups, with PE formulations producing ~ 14-fold increases and DMG a more modest 6-fold (Fig. 3D). PE-PEG750 caused earlier increases in total IgG compared to the other formulations. Anti-PEG IgG responses were similar, with DMG-PEG2000 producing a slightly stronger induction (7-fold vs. 4-fold for PE PEG-lipids, Fig. 3E). Despite large differences in antibody magnitude across PEG-lipid chemistries, efficacy loss was invariant, indicating that PEG-associated clearance occurs once a threshold level of anti-PEG IgM is exceeded.

These data suggest that PEG-lipid chemistry, particularly linker identity, modulates the magnitude of the adaptive immune response. This finding complements our recent demonstration that ionizable lipid chemistry drives innate immune sensing through TLR4 and CD1d [30]. Taken together, these studies indicate that modifying the PEG component cannot compensate for an ionizable lipid that fails to engage innate immune receptors. Because the ionizable lipid governs whether pro-inflammatory cytokines suppress anti-PEG IgM production, the identity of the stealth polymer is secondary to the immunological profile of the ionizable core. Nevertheless, alternative stealth polymers such as polysarcosine[42, 43] or brush polymers[44] may reduce anti-polymer antibody responses, though they do not address the underlying immunological role of the ionizable lipid[31].

### PEG-lipid concentrations required for delivery induce anti-PEG antibodies

Because PEG-lipid concentration directly controls colloidal stability, blood clearance, and the density of PEG epitopes available for immune recognition, we next asked whether reducing PEG mol% could mitigate adaptive immune responses without compromising delivery. To assess this, 304O_i10_ LNPs were formulated with PE-PEG2000 at commonly used concentrations from 0 to 2.5 mol% in 0.5% increments[41, 45]. These formulations were IV-injected twice 14 days apart (Scheme 1).

**Figure 4:**
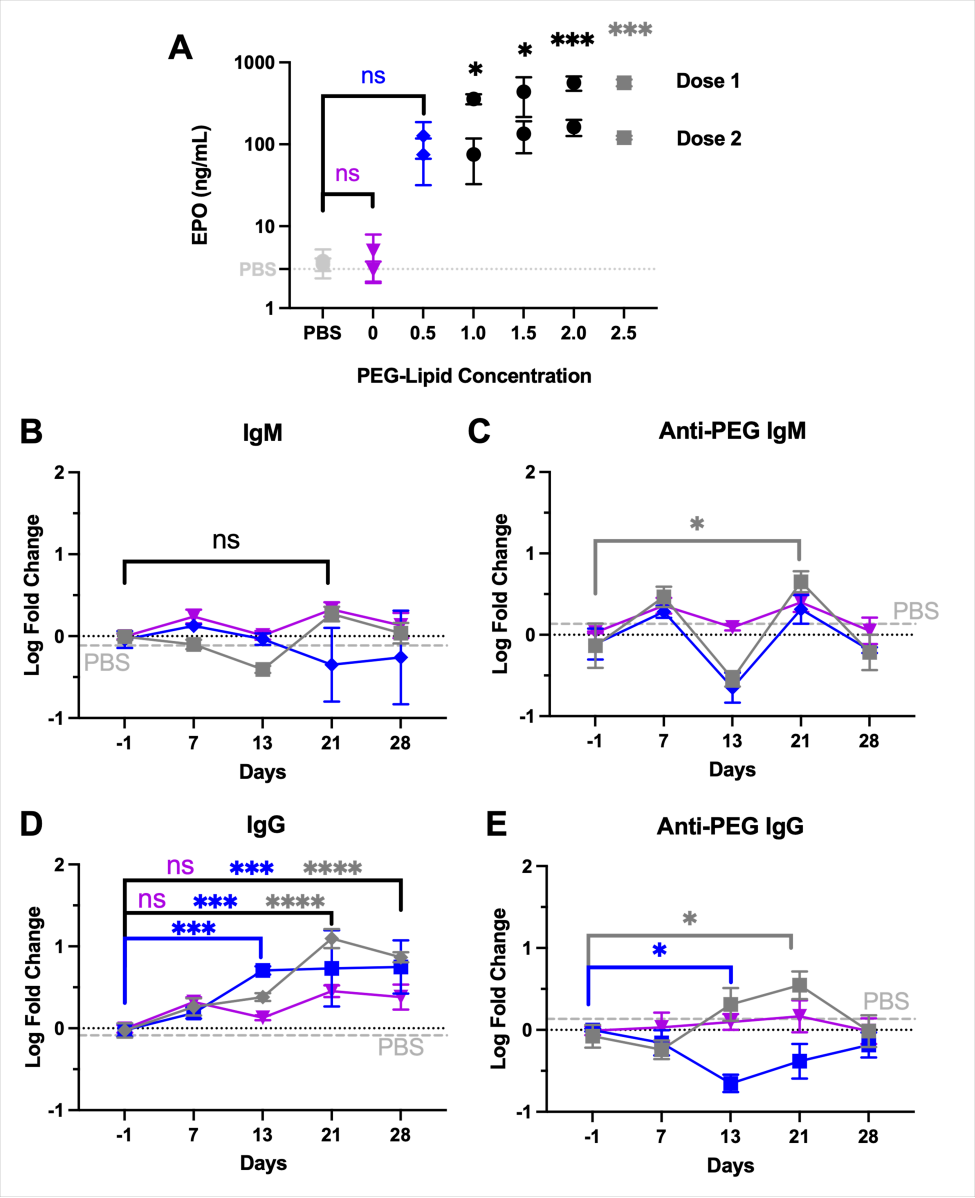
PEG formulation concentrations that are required for efficacy also induce anti-PEG antibody production. Lipid nanoparticles formulated with 304O_i10_ and PE-PEG2000 were delivered IV to mice at 0.5 mg/kg with mRNA encoding EPO. (A) LNPs were formulated with 0% (purple triangles), 0.5% (blue diamonds), and 2.5% (gray squares) PEG concentrations and dosed twice to mice 14 days apart. Serum EPO concentrations were quantified by ELISA 6 hours after each dose, and efficacy decreased with each dose. (B-E) Serum antibody levels were quantified over time by ELISA. Anti-PEG IgM and total IgG increased significantly for formulations with ≥1.0% PEG. Anti-PEG IgG increased significantly for 2.5% PEG. Antibody levels for 1.0–2.0% PEG concentrations are shown in Fig S4. The gray dashed line indicates the serum levels of the PBS control group. N=3–4, error bars represent SEM, with * and ***, **** indicating P < 0.05, and P < 0.001, P < 0.0001, respectively, by two-way ANOVA.

As shown in Fig. 4A, formulations with ≥ 1.0% PEG enabled robust EPO expression but showed clear efficacy loss upon redosing. Formulations with ≤ 0.5% PEG showed no measurable efficacy loss, but they also produced EPO levels indistinguishable from PBS controls. In other words, there was no efficacy to lose. These data reveal a threshold effect: sufficient PEG is required for efficacy, but any concentration that supports expression also triggers adaptive immunity. Expression levels correlated positively with PEG mol% (R^2^ = 0.93, **Fig S3**), consistent with the role of PEG-lipids in maintaining LNP colloidal stability and slowing clearance from the bloodstream.

Antibody profiling supported the efficacy-immunogenicity tradeoff. Although total IgM levels remained unchanged across all groups from day – 1 to 21 (Fig. 4B, S4A), anti-PEG IgM antibody expression rose significantly only for formulations achieving statistically significant EPO expression (≥ 1.0% PEG), with fold increases ranging from 3 to 31 (Fig. 4C, S4B). Total IgG rose significantly for the same formulations (Fig. 4D, S4C), likely reflecting responses to the ionizable lipid or RNA [26, 30]. Only the 2.5% PEG formulation induced a significant rise in anti-PEG IgG on day 21, suggesting that a higher PEG surface density is required to drive class switching beyond IgM (Fig. 4E, S4D). Together, these results indicate an efficacy-immunogenicity tradeoff governed by PEG concentration.

### Anti-PEG immunity impairs cross-formulation LNP dosing

Patients receiving RNA therapeutics may encounter multiple PEGylated LNP products over time, whether through sequential therapies or different vaccine platforms. A critical question is therefore whether anti-PEG responses elicited by one formulation can compromise subsequent treatment with a different LNP formulation. To investigate this, we examined mRNA delivery efficacy following sequential dosing of 304O_i10_ LNPs and LNPs formulated with the FDA-approved ionizable lipid, DLin-MC3-DMA (MC3). MC3 is the ionizable lipid in the first FDA-approved lipid nanoparticle drug, patisiran, which is successfully repeat-dosed in the clinic[46, 47]. In these experiments, all LNP formulation parameters were held constant except the ionizable lipid chemistry. This choice minimizes any potential confounding effects of molar ratio choices or PEG and helper lipid identity.

**Figure 5:**
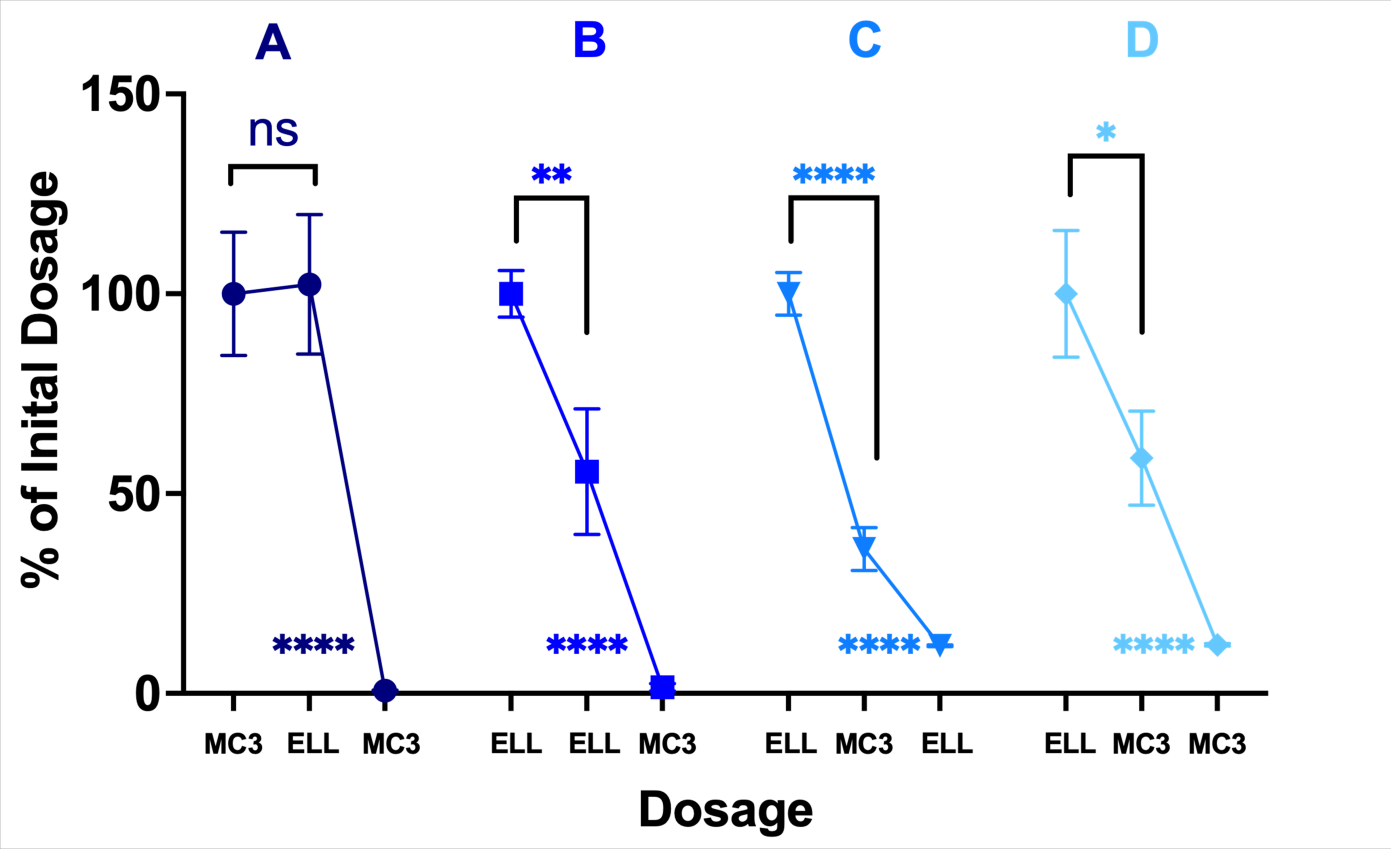
Pre-existing anti-PEG antibodies inhibit de novo efficacy of an FDA-approved ionizable lipid. Lipid nanoparticles formulated with either MC3 or 304O_i10_ were delivered IV to mice at 0.5 mg/kg with EPO mRNA for three treatment regimens. A) When an initial dose of MC3 LNPs was followed by 304O_i10_ LNPs, efficacy was retained, but a subsequent MC3 dose lost efficacy. (B-D) When 304O_i10_ LNPs were dosed first, followed by 304O_i10_ or MC3 LNPs, a significant drop in efficacy was observed. N=3–4, error bars represent SEM, with * and **, **** indicating P < 0.05, P < 0.01, and P < 0.0001, respectively, by two-way ANOVA.

As a validation step, we first confirmed that MC3 LNPs produced robust EPO expression following two IV administrations at a 14-day interval (**Fig S5**). We then conducted repeat dosing experiments for four formulation scenarios (A-D), each of which involved 3 doses of EPO mRNA: MC3–304-MC3, 304–304-MC3, 304-MC3–304, and 304-MC3-MC3. Figure 5 shows that efficacy for either formulation is always compromised following a dose of 304O_i10_ LNPs. Notably, the third dose of formulation schemes B-D always presented additional drops in EPO expression over dose two, regardless of ionizable lipid chemistry. Formulation D, in particular, demonstrates that the formation of anti-PEG antibodies following a first dose drives the overall loss-of-efficacy phenomenon. The second injection, in this case MC3, essentially serves as a booster for anti-PEG immunogenicity.

These results demonstrate that anti-PEG antibodies elicited by an LNP containing an efficacy-loss-inducing ionizable lipid can compromise the performance of other PEGylated LNPs, including those based on FDA-approved ionizable lipids. This cross-formulation interference implies that the anti-PEG antibodies recognize the PEG backbone itself rather than specific features of the PEG-lipid linker or surface density, consistent with the persistence of efficacy loss across all PEG-lipid chemistries and concentrations tested above. Given that ~ 25% of people already carry anti-PEG antibodies [48], probably due to widespread exposure through consumer products, pharmaceuticals, and processed foods [48–50], this effect may be even more pronounced in humans than in naïve mice. Although some clinical studies suggest PEG allergy is not universally prohibitive for vaccination[51, 52], variability in PEG type and molecular weight complicates prediction.

Together, these findings underscore the importance of evaluating candidate LNPs not only for standalone repeat dosing performance but also for their capacity to interfere with subsequent nanoparticle therapies. Future preclinical pipelines should incorporate repeat dosing studies in the context of prior PEG exposure, as real-world patients will present with heterogeneous baseline antibody levels. Together with our prior demonstration that 304O_i10_ fails to engage TLR4 and CD1d, these data establish a two-part picture: ionizable lipids that bypass innate immune sensing permit anti-PEG IgM production, and the resulting antibodies then compromise any PEGylated formulation administered subsequently, including those built on FDA-approved ionizable lipids. The implication is that ionizable lipid selection determines repeat dosing success, and PEG-side modifications cannot rescue an ionizable lipid that fails at the innate immune step.

These data also raise the question of whether PEG alternatives, such as polysarcosine or poly(2-oxazoline)s, would similarly become targets of anti-polymer antibodies when formulated with ionizable lipids, such as 304O_i10_, that lack innate immune receptor engagement. Our results suggest that the immunological vulnerability lies upstream of the stealth polymer, driven by the ionizable lipid’s failure to suppress B cell-mediated antibody production. If so, simply substituting PEG with another polymer may shift the antibody target without eliminating the underlying problem.

## Conclusion

These studies establish that PEG-associated efficacy loss persists across variations in PEG-lipid chemistry, PEG concentration, mRNA cargo, and administration route. Moreover, anti-PEG immunity established by 304O_i10_ LNPs compromised the subsequent performance of MC3 LNPs, demonstrating that this is not a formulation-specific defect but a fundamental consequence of PEG-directed adaptive immunity. These findings are consistent with our previous work showing that the ionizable lipid’s ability to engage innate immune receptors determines whether pro-inflammatory cytokines suppress anti-PEG IgM production. When the ionizable lipid lacks this innate immune engagement, as is the case for 304O_i10_, no downstream PEG-lipid modification can compensate. Together with those earlier findings, the present data establish that the path to durable repeat dosing requires addressing the immunological interface between LNPs and the innate immune system, whether through selection of ionizable lipids that actively suppress anti-PEG responses, development of truly PEG-free delivery platforms, and/or targeted modulation of the immune pathways that govern anti-polymer antibody production.

## Methods

### Materials.

The amine N1-methyl-N2,N2-bis(2-(methylamino)ethyl)ethane-1,2-diamine (304) was purchased from Sigma Aldrich. Isodecyl acrylate (O_i10_) was sourced from Sartomer (Colombes, France). DLin-MC3-DMA (**MC3**) was purchased from BroadPharm (San Diego, CA). Lipid nanoparticle PEG-lipid and helper lipid components were purchased from Avanti Polar Lipids: 1,2-dioleoyl-sn-glycero-3-phosphoethanolamine (**DOPE**); 1,2-dimyristoyl-sn-glycero-3-phosphoethanolamine-N-[methoxy(polyethyleneglycol)-2000] (ammonium salt) (**PE-PEG 2000 MW**); 1,2-dimyristoyl-sn-glycero-3-phosphoethanolamine-N-[methoxy(polyethyleneglycol)-750] (ammonium salt) (**PE-PEG 750 MW**); and 1,2-dimyristoyl-rac-glycero-3-methoxypolyethyleneglycol-2000 (**DMG-PEG 2000 MW**). Cholesterol was purchased from Sigma Aldrich. All mRNAs used here were a generous gift from Translate Bio (now part of Sanofi).

### Synthesis of Ionizable Lipids.

As previously described, ionizable lipids were synthesized by reacting alkyl-acrylates with alkyl-amines using Michael addition chemistry[53, 54]. Specifically, the amine 304 was reacted with the alkyl-acrylate O_i10_, at a molar ratio of 3.7:1 at 90°C for 3 days under constant stirring. After synthesis, the crude mixture was purified through flash chromatography using a CombiFlash Rf 200 (Teledyne ISCO, Lincoln, NE), and fractions corresponding to different lipids were isolated. Lipid structure of different fractions was verified using NMR, and the fraction corresponding to 304O_i10_ was stored at 4°C till use.

### Formation of Lipid Nanoparticles.

LNPs were formulated by mixing ionizable lipid, cholesterol, DOPE, and PE-PEG 2000 MW C14, in a solution of ethanol and 10 mM sodium citrate 9:1 by volume at a molar ratio of 35:46.5:16:2.5. mRNA was diluted in 10 mM sodium citrate buffer. Lipid and mRNA solutions were combined to form nanoparticles through flash nanoprecipitation[54]. LNPs had a final weight ratio of 1:10 mRNA to ionizable. LNPs were diluted to the desired concentration using phosphate buffered saline (PBS). For experiments examining the role of PE-PEG 2000 MW molar percentage, the molar ratio of ionizable : cholesterol : DOPE : PE-PEG 2000 MW was 35 : 49-X : 16 : X.

### In Vivo Testing of Lipid Nanoparticles.

All animal experiments were conducted under an institutionally approved protocol approved by the Institutional Animal Care and Use Committee at Carnegie Mellon University. C57BL/6 mice, 6–8 weeks in age, were used for all experiments. Mice were injected with mRNA-LNPs encoding firefly luciferase (fLuc) or human Erythropoietin (hEPO) through intravenous, intramuscular, or intraperitoneal routes at a dose of 0.5 mg/kg. hEPO levels were measured using ELISA (described below). For measuring luciferase levels, mice were intraperitoneally injected with 130 μL of D-luciferin (30 mg/kg, PerkinElmer). Fifteen minutes later, mice were euthanized, and their organs were excised and imaged using *In Vivo* Imaging Instruments (IVIS, Perkin Elmer). Luminescent intensity was quantified using Region of Interest analysis in Living Image software.

### Human Erythropoietin ELISAs.

Human Erythropoietin levels were measured using a Human Erythropoietin/EPO Quantikine ELISA Kit, RUO (R&D Systems, Minneapolis, MN) as per the manufacturer’s instructions. Blood samples were centrifuged and recovered serum was diluted 1:1500 for ELISA. The optical density of each well was measured at 450/570 nm using a Synergy H1 microplate reader (BioTek Instruments, Winooski, VT).

### Immunoglobulin ELISA.

IgM and IgG levels were measured using the C57BL/6-HRP clonotyping kit (Southern Biotech, Birmingham, AL) as per the manufacturer’s instructions. For anti-PEG ELISAs, 100 μg/mL PEG-lipid was dissolved in PBS and coated overnight on high binding polystyrene plates (Corning). Plates were washed twice with 0.05% v/v Tween 20 in PBS, and incubated with 5% FBS in PBS for at least one hour. Serum samples were diluted 1:200 for anti-PEG antibodies and 1:10,000 for total IgG and IgM. They were incubated for at least two hours at room temperature. To create a standard curve, anti-PEG IgM (clone ANPEG-1, ANP Technologies, Newark, DE) and anti-PEG IgG (clone 1D9–6, Life Diagnostics, West Chester, PA) were used. Plates were washed twice and incubated for at least one hour with 1 μg/mL goat anti-mouse IgG-HRP or goat anti-mouse IgM-HRP. Plates were washed four times, and 100 μL TMB was added to each well and incubated in the dark for 3–5 min. The reaction was stopped by adding 100 μL 1M H_2_SO_4_. The optical density of each well was measured at 450/570 nm using a Synergy H1 microplate reader (BioTek Instruments, Winooski, VT).

### Statistical Analysis.

All data were processed using GraphPad Prism. ELISA data for IgG, IgM, and anti-PEG were normalized to the average of the serum level of the protein before experiments occurred (day – 1). Additionally, these ELISA data were not normally distributed, and therefore, a log transform was performed. Analysis of Variance (ANOVA) was used for statistical tests on normalized and log-transformed data, and post-hoc Tukey's multiple comparisons test was used.

## Supplementary Material

Supplementary Files

This is a list of supplementary files associated with this preprint. Click to download.

• 260519RepeatDoseSupFiguresvSubmit.docx

• GraphicalAbstract.docx

• RDScheme1.png

Scheme 1 is available in the Supplementary Files section.

## Data Availability

The datasets from this study are available from the corresponding author upon reasonable request.
